# Olive (*Olea europaea* L.) Seed as New Source of Cholesterol-Lowering Bioactive Peptides: Elucidation of Their Mechanism of Action in HepG2 Cells and Their Trans-Epithelial Transport in Differentiated Caco-2 Cells

**DOI:** 10.3390/nu16030371

**Published:** 2024-01-26

**Authors:** Martina Bartolomei, Jianqiang Li, Anna Laura Capriotti, Melissa Fanzaga, Lorenza d’Adduzio, Aldo Laganà, Andrea Cerrato, Nadia Mulinacci, Lorenzo Cecchi, Carlotta Bollati, Carmen Lammi

**Affiliations:** 1 Department of Pharmaceutical Sciences, University of Milan, 20133 Milan, Italy; martina.bartolomei@unimi.it (M.B.); melissa.fanzaga@unimi.it (M.F.); lorenza.dadduzio@unimi.it (L.d.);; 2 Department of Chemistry, Sapienza University of Rome, Piazzale Aldo Moro 5, 00185 Rome, Italyaldo.lagana@uniroma1.it (A.L.); andrea.cerrato@uniroma1.it (A.C.); 3 Department of Neuroscience, Psychology, Drug and Child Health, Pharmaceutical and Nutraceutical Section, University of Florence, 50019 Florence, Italy; nadia.mulinacci@unifi.it; 4 Department of Agricultural, Food, Environmental and Forestry Sciences and Technologies, University of Florence, Via Donizetti, 50144 Florence, Italy; lo.cecchi@unifi.it

**Keywords:** LDLR, cholesterol metabolism, food bioactive peptides, multifunctional peptides, PCSK9

## Abstract

The production of olive oil has important economic repercussions in Mediterranean countries but also a considerable impact on the environment. This production generates enormous quantities of waste and by-products, which can be exploited as new raw materials to obtain innovative ingredients and therefore make the olive production more sustainable. In a previous study, we decided to foster olive seeds by generating two protein hydrolysates using food-grade enzymes, alcalase (AH) and papain (PH). These hydrolysates have shown, both in vitro and at the cellular level, antioxidant and antidiabetic activities, being able to inhibit the activity of the DPP-IV enzyme and modulate the secretion of GLP-1. Given the multifunctional behavior of peptides, both hydrolysates displayed dual hypocholesterolemic activity, inhibiting the activity of HMGCoAR and impairing the PPI of PCSK9/LDLR, with an IC_50_ equal to 0.61 mg/mL and 0.31 mg/mL for AH and PH, respectively. Furthermore, both samples restored LDLR protein levels on the membrane of human hepatic HepG2 cells, increasing the uptake of LDL from the extracellular environment. Since intestinal bioavailability is a key component of bioactive peptides, the second objective of this work is to evaluate the capacity of AH and PH peptides to be transported by differentiated human intestinal Caco-2 cells. The peptides transported by intestinal cells have been analyzed using mass spectrometry analysis, identifying a mixture of stable peptides that may represent new ingredients with multifunctional qualities for the development of nutraceuticals and functional foods to delay the onset of metabolic syndrome, promoting the principles of environmental sustainability.

## 1. Introduction

The Mediterranean basin’s countries greatly benefit economically from the production of olive oil. However, its production is associated with a serious environmental impact due to resource depletion, soil degradation, and the generation of enormous quantities of waste and by-products. These negative effects can vary depending on the usage and techniques used both during cultivation and during the extraction of oil from olives [1,2]. Therefore, the use of innovative cultivation and oil extraction techniques can bring about economic, environmental, and social transformations. For instance, the pitting phase could be a simple but effective answer from both an economic and environmental point of view. The pitting step makes it possible to obtain a paste (oil pomace) from which the oil extraction yield is higher, and the by-product, the olive stone, is recovered and is easily accessible, as it does not require any additional processing to be used [3]. This by-product is highly valued for energy use and has better combustion efficiency and a low ash content in comparison with the pomace [4]. In a previous work, we decided to valorize this by-product to obtain new and higher value-added products and to achieve more sustainable and profitable production in the olive oil sector. In detail, we targeted the extraction and characterization of the proteins present in the olive seeds, derived from the *Frantoio* cultivar of *Olea europaea* L., to generate hydrolysates rich in bioactive peptides using two food-grade enzymes, i.e., alcalase and papain, obtaining AH and PH samples, respectively. About 104 medium and 491 short peptides were identified within the hydrolysates, and it was also demonstrated their antioxidant and antidiabetic properties by investigating the inhibition of the dipeptidyl peptidase IV (DPP-IV) enzyme and modulation of incretin hormone glucagon-like peptide-1 (GLP-1) levels using a combination of in vitro and cellular assays [5]. Considering that protein hydrolysates often show multifunctional behavior due to their heterogeneous composition and the ability of peptides to interact with two or more biological pathways [6,7,8], the first objective of this work was to study new possible biological activities of AH and PH, focusing on the hypocholesterolemic one, and evaluate their effects in modulating the cholesterol metabolism. Notably, the rate-limiting enzyme involved in the intracellular generation of cholesterol is 3-hydroxy-3-methylglutaryl coenzyme A reductase (HMGCoAR) [9]. When this enzyme is inhibited, a reduction in intracellular cholesterol biosynthesis occurs, leading to the activation of the sterol regulatory element-binding protein (SREBP)-2 transcription factor, which, in order to maintain cholesterol homeostasis, improves the low-density lipoprotein receptor (LDLR) and HMGCoAR (its two main target) protein levels. The improved LDLR on the surface of hepatocytes is correlated with an improved ability of hepatic cells to clear plasmatic LDL-cholesterol, which ultimately results in a hypocholesterolemic effect [10,11,12].

Given the constant increase in the incidence of cardiovascular diseases (CD), there has been a growing interest in strategies capable of reducing their onset by modifying lifestyle and diet [13]. Peptides, especially those derived from soy and lupin, which show hypocholesterolemic effects, have been reported in the literature. The mechanisms of the action involved are multiple, such as increasing the expression of the low-density lipoprotein receptor (LDLR), reducing the absorption of sterols at the intestinal level, and increasing the secretion of bile acids [14,15,16,17]. Over the last decade, peptides capable of inhibiting the proprotein convertase subtilisin/kexin type 9 (PCSK9), responsible for LDLR degradation in the liver, have also been identified [18]. PCSK9 is regulated by its N-terminal prodomain, which is enzymatically cleaved for protein activation and acts by binding to the EGF-A domain of LDLR on the cell surface. Dietary peptides that structurally resemble the LDLR prodomain or EGF-A domain could be investigated as PCSK9 ligands and inhibitors [17,19]. Peptides with inhibitory activity on the PCSK9 protein stimulate the absorption of cholesterol by the LDLR, with a consequent increase in its degradation in hepatocytes. The current work assesses the AH and PH hypocholesterolemic activity through the modulation of the HMGCoAR enzyme and PCSK9/LDLR protein–protein interaction (PPI) in human hepatic HepG2.

A problem that many protein hydrolysates present is the lack of stability in the extensive metabolism phase that occurs in the gastrointestinal tract and, therefore, poor bioavailability. Therefore, bioavailability is the limiting factor for the application of protein hydrolysates. Peptides that show resistance to the digestive enzymes’ activity in the gastrointestinal tract and to peptidases present in the brush border membrane have a greater chance of being absorbed through the intestine [20]. Indeed, although several peptides have been identified as having multiple beneficial effects on human health, there is little evidence of their bioavailability [21]. A widely used model to simulate the human intestine in terms of biological functions and structure is the Caco-2 cell monolayer obtained from human intestinal carcinoma. In fact, this monolayer is widely used to study drug absorption across the epithelium since differentiated cells express all the major digestive enzymes and transport proteins present in the human small intestine, providing metabolism as well as active and passive transport to be examined [22,23]. The absorption of peptides derived from olive seeds has never been evaluated; hence, the second aim of this work was to investigate the AH and PH in vitro trans-epithelial transport across differentiated human intestinal Caco-2 cell monolayers.

## 2. Materials and Methods

### 2.1. Chemicals

All chemicals and reagents were commercially available, and additional information is reported in the Supplementary Materials.

### 2.2. Sample Preparation

Alcalase and papain olive seed hydrolysates were prepared as previously described [5]. Briefly, after extraction from the olive seed, proteins were hydrolyzed with alcalase (50 °C, 4 h, 0.15 UA/g, pH 8.5) and papain (65 °C, 8 h, 100 UA/g, pH 7) enzymes (Sigma Aldrich, Milan, Italy). Both hydrolysates were ultrafiltrated with a 3 kDa cut-off Millipore UF System ultrafiltration membrane using optimized conditions (Sigma Aldrich, Milan, Italy) [7].

### 2.3. Cell Culture

Human intestinal Caco-2 cells INSERM (Paris, France) and human hepatic HepG2 cells (ATCC, HB-8065, ATCC from LGC Standards, Milan, Italy) were cultured in DMEM high glucose with stable L-glutamine, supplemented with 10% FBS, 100 U/mL penicillin, and 100 μg/mL streptomycin (complete growth medium). The cultures were incubated at 37 °C in a 5% CO_2_ atmosphere.

### 2.4. 3-(4,5-Dimethylthiazol-2-yl)-2,5-Diphenyltetrazolium Bromide (MTT) Assay

A total of 3 × 10^4^ HepG2 cells/well were seeded in 96-well plates and treated with 0.1, 0.5, 1.0, 1.5, and 5.0 mg/mL of AH and PH samples or vehicle (H_2_O) in complete growth media for 48 h at 37 °C under a 5% CO_2_ atmosphere. MTT studies were conducted in pre-optimized settings [24]. More details are available in the Supplementary Materials.

### 2.5. In Vitro PCSK9-LDLR Binding Assay

The in vitro PCSK9-LDLR binding assay (CycLex Co., Nagano, Japan) was used to test AH and PH (1 mg/mL) in accordance with the manufacturer’s instructions and under pre-optimized conditions [25].

### 2.6. In-Cell Western (ICW) Assay

The ICW assay was performed using the same previously optimized procedure [26]. Briefly, a total of 3 × 10^4^ HepG2 cells/well were seeded in a 96-well plate, and the following day, they were treated with 4.0 µg/mL PCSK9-WT, 1 mg/mL of AH and PH, 4.0 µg/mL PCSK9 + 1 mg/mL of AH and PH, and vehicle (H_2_O) for 2 h at 37 °C under a 5% CO_2_ atmosphere. Subsequently, the ICW assay was performed in accordance with the protocol that is available in the Supplementary Materials.

### 2.7. Fluorescent LDL Uptake

The LDL uptake assay was performed following the conditions already described [27]. In brief, 3 × 10^4^ HepG2 cells/well were seeded and then kept in complete growth medium for 2 d prior to treatment. On the third day, cells were treated with 4.0 µg/mL PCSK9-WT, 1 mg/mL of AH and PH, 4.0 µg/mL PCSK9 + 1 mg/mL of AH and PH, and vehicle (H_2_O) for 2 h at 37 °C under a 5% CO_2_ atmosphere, and LDL-uptake was carried out following the protocol that is detailed in the Supplementary Materials.

### 2.8. Western Blot Analysis

Immunoblotting experiments were carried out with an optimized technique [28]. 1.5 × 10^5^ HepG2 cells/well (24-well plate) were treated with 1 mg/mL of AH and PH for 24 h. More details are available in the Supplementary Materials.

### 2.9. HMGCoAR A Activity Assay

The experiments were carried out in accordance with the manufacturer’s guidelines and the recommended protocol [29]. More details are available in the Supplementary Materials.

### 2.10. Caco-2 Cell Culture and Differentiation

Caco-2 cells were cultured as reported by a previous protocol [30]. Additional information is provided in the Supplementary Materials.

### 2.11. Trans-Epithelial Transport Experiments

TEER measurement was used to verify the integrity and differentiation of the cell monolayer prior to investigations. Hydrolysate trans-epithelial passage was assayed in differentiated Caco-2 cells in transport buffer solution (137 mM NaCl, 5.36 mM KCl, 1.26 mM CaCl_2_, and 1.1 mM MgCl_2_, 5.5 mM glucose) in accordance with previously described conditions [31]. More details are reported in the Supplementary Materials.

### 2.12. UHPLC-HRMS Analysis and Short-Sized Peptide Identification

Short peptides were analyzed by Vanquish binary pump H (Thermo Fisher Scientific, Str. Rivoltana—Rodano, Milan, Italy) coupled to a hybrid quadrupole—Orbitrap mass spectrometer Q Exactive (Thermo Fisher Scientific, Str. Rivoltana—Rodano, Milan, Italy) using a heated ESI source operating in positive ion mode. The mass-spectrometric strategy was developed as previously documented [32]. More details are reported in the Supplementary Materials.

### 2.13. In Silico Toxicity Prediction of the Bioavailable Fraction of AH and PH Hydrolysates

The peptides, which were identified as the most abundant in the BL side of the Transwell system exploited for the absorption studies for both AH and PH hydrolysates, were analyzed using a web-based server Toxinpred (https://webs.iiitd.edu.in/raghava/toxinpred/multi_submit.php (accessed on 3 January 2024)), which is useful to identify and predict highly toxic or non-toxic peptides from a large number of submitted sequences in FASTA format [33].

### 2.14. Statistical Analysis

Every measurement was performed in triplicate, and results were expressed as the mean ± standard deviation (s.d.), where *p*-values < 0.05 were considered to be significant. Statistical analyses were performed by one-way ANOVA followed by Dunnett’s and Tukey’s post-test (Graphpad Prism 9, GraphPad Software, La Jolla, CA, USA).

## 3. Results

### 3.1. Olive Seed Peptides Target PCSK9/LDLR PPI and HMGCoAR Activity with a Dual Inhibitory Effect

#### 3.1.1. Alcalase (AH) and Papain (PH) Hydrolysates Impairs the PCSK9/LDLR PPI

**Figure 1:**
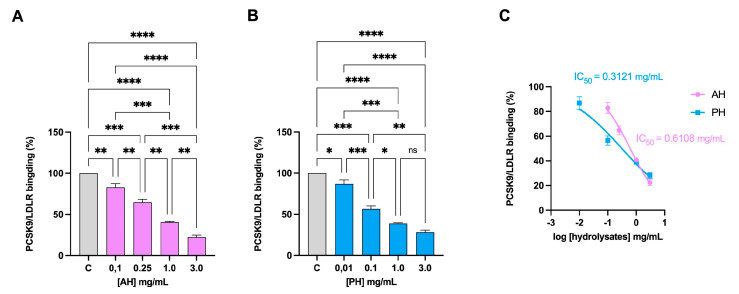
Inhibition of the PPI between PCSK9 and LDLR. AH (**A**) and PH (**B**) impairment of the protein–protein interaction between PCSK9 and LDLR with a dose-dependent trend. PCSK9/LDLR binding and IC_50_ assessment (**C**). Data represent the mean ± s.d. of three independent experiments performed in triplicate. Data were analyzed by one-way ANOVA followed by Tukey’s post-hoc test; (*) *p* < 0.05; (**) *p* < 0.01; (***) *p* < 0.001; (****) *p* < 0.0001. ns: not significant. C: control sample.

The LDL receptor interacts with a protein produced in several organs, such as the liver, kidneys, and intestines, named PCSK9. This interaction causes the activation of the catabolism of the receptor, leading to its degradation, especially in the liver [34]. We therefore evaluated whether the hydrolysates were able to inhibit this interaction. Both AH and PH can reduce PCSK9-LDLR binding with a dose–response trend (Figure 1A,B) with IC_50_ of 0.61 mg/mL and 0.31 mg/mL, respectively (Figure 1C).

#### 3.1.2. Olive Seed Hydrolysates Drop In Vitro the HMGCoAR Activity

**Figure 2:**
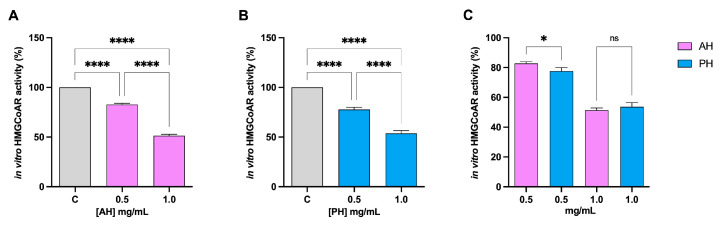
Effect of AH and PH on the modulation of in vitro HMGCoAR activity. Both AH (**A**) and PH (**B**) hydrolysates drop HMGCoAR with dose–response trends. Comparison between the enzymes (**C**). Data represent the mean ± s.d. of six independent experiments performed in triplicate. Data were analyzed by one-way ANOVA followed by Tukey’s post-hoc test; (*) *p* < 0.05; (****) *p* < 0.0001. ns: not significant. C: control sample.

The HMGCoAR enzyme is considered the rate-limiting enzyme in the intracellular biosynthesis of cholesterol, and it is the target of statins, the primary drugs used to treat hypercholesterolemia [35]. Hence, the potential HMGCoAR inhibitory activity of each hydrolysate was assessed by further biochemical investigation. The data presented in Figure 2 unequivocally imply that both hydrolysates decrease enzyme activity with dose–response trends. At concentrations of 0.5 and 1 mg/mL, for the AH hydrolysate, the residual activity is 82.7 ± 1.2% and 51.3 ± 1.5%, respectively (Figure 2A), whereas for the PH hydrolysates, it is 77.65 ± 2.3% and 53.7 ± 2.7%, respectively (Figure 2B). Comparing hydrolysates produced with different enzymes, no significant difference is observed at a concentration of 1 mg/mL (Figure 2C).

### 3.2. Assessment of Olive Seed Peptide Ability to Modulate the Cholesterol Metabolism in HepG2 Cells

#### 3.2.1. Alcalase (AH) and Papain (PH) Hydrolysates Do Not Show Any Cytotoxic Effect on HepG2

**Figure 3:**
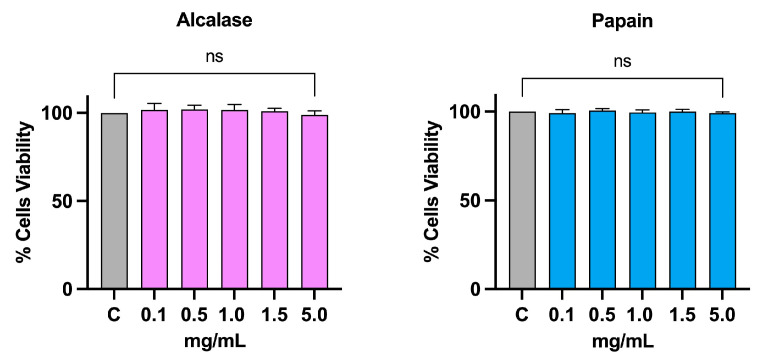
Cell vitality after treatment with AH and PH. Both hydrolysates do not affect the HepG2 vitality after 48 h of incubation up to 5 mg/mL. Data represent the mean ± s.d. of six independent experiments performed in triplicate. Data were analyzed by one-way ANOVA followed by Dunnett post-hoc test. ns: not significant. C: control sample.

The possible cytotoxic effect of alcalase (AH) and papain (PH) hydrolysates on intestinal cells Caco-2 had already been evaluated in a previous study. Treatment up to 5 mg/mL did not show any toxicity [5]. Before proceeding with the experiments on hepatic HepG2 cells, an MTT was conducted to exclude potential cytotoxic effects in this cell line. As shown in Figure 3, increasing concentrations of AH and PH (0.1, 0.5, 1, 1.5, and 5 mg/mL) were tested, and following 48 h of treatment, no appreciable cell mortality was observed at any of the tested doses as compared to the vehicle (H_2_O).

#### 3.2.2. Alcalase (AH) and Papain (PH) Hydrolysates Modulate the Hepatic LDLR Pathway in Human Hepatic HepG2 Cells

**Figure 4:**
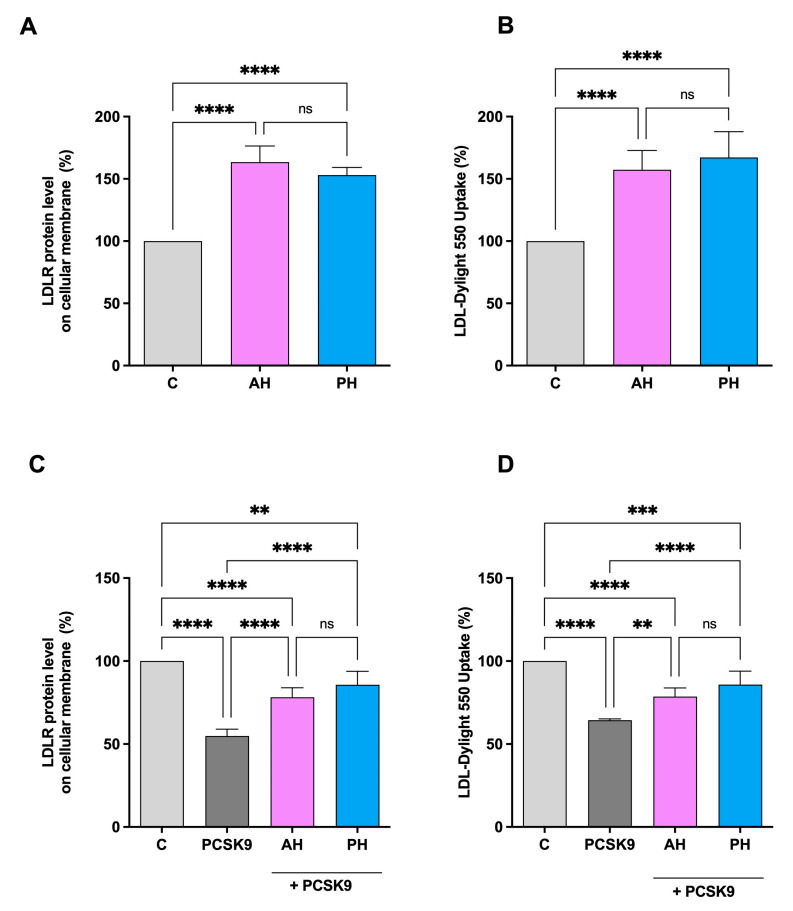
Effects of PH and AH on LDLR protein level and activity specifically located on the surface of hepatic cells. HepG2 cells were treated with AH and PH (1 mg/mL) for 24 h. The percentage of LDLR protein level was measured by ICW assay (**A**). The treatment of HepG2 cells with PCSK9 (4 μg/mL) reduced active LDLR protein levels localized on the surface of cells, which were restored by both AH and PH (**B**). Effect of AH and PH (1 mg/mL) on the HepG2 cell ability to uptake LDL from extracellular environment (**C**). Following PCSK9 (4 μg/mL) incubation, HepG2 cells’ functional capacity to absorb LDL from the extracellular space was diminished; in contrast, after treatment with both hydrolysates, it increased (**D**). Data represent the mean ± s.d. of three independent experiments performed in triplicate. Data were analyzed by one-way ANOVA followed by Tukey’s post-hoc test; (**) *p* < 0.01; (***) *p* < 0.001, (****) *p* < 0.0001. ns: not significant. C: control sample.

The ICW assay is a quantitative colorimetric cell-based assay that allows the identification of target proteins in fixed cultured cells. It was used to evaluate the ability of AH and PH to modulate the protein level of LDLR on the HepG2 cell surface [31]. As seen in Figure 4A, there is an improvement in the level of LDLR specifically localized on the cell membrane of hepatocytes, up to 163.4 ± 13.1% for and up to 153.1 ± 6.1%. The results are also confirmed by repeating the experiment in the presence of PCSK9 [26]. In fact, treatment with PCSK9 alone leads to a significant reduction of 45.17 ± 4.1% in the receptor levels, while we only have a reduction of 21.8 ± 5.7% and 14.24 ± 8.6% compared to untreated cells for AH and PH, respectively (Figure 4C). In both experiments, no significant difference was observed between AH and PH. The increase in membrane LDLR protein levels resulted in an enhanced functional ability of HepG2 cells to absorb extracellular LDL by 157.3 ± 15.4% and 167.2 ± 20.7% after treatment with AH and PH at the same concentration of 1 mg/mL (Figure 4B). Following the treatment with PCSK9 alone, the capacity of HepG2 cells to uptake fluorescent LDL was lower by 35.66 ± 0.8% in contrast to untreated cells, and the treatment with AH and PH reversed this effect up to 78.59 ± 5.2% and 85.8 ± 8.0%, as shown in Figure 4D. Also, in this case, we did not observe significant differences between the two hydrolysates.

**Figure 5:**
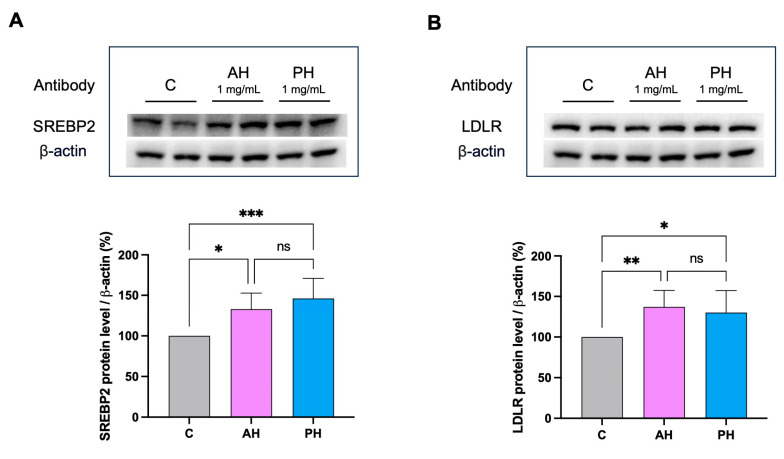
In HepG2 cells treated with AH and PH, the LDLR pathway is modulated. After the treatment of HepG2 cells with AH and PH, the SREBP-2 protein level was enhanced (**A**) as well as (**B**). Data points represent the averages ± SD of four independent experiments performed in duplicate. Data were analyzed by one-way ANOVA followed by Tukey’s post-hoc test; (*) *p* < 0.05; (**) *p* < 0.01 (***) *p* < 0.001. ns: not significant. C: untreated HepG2 cells.

The activation of the receptor was also confirmed by immunoblotting experiments where HepG2 cells were treated for 24 h with AH and PH at a concentration of 1 mg/mL. More in detail, AH and PH hydrolysates up-regulate the protein levels of SREBP-2 transcription factor by 132.9 ± 19.8% and 146.0 ± 24.8%, respectively (Figure 5A). The increase in SREBP-2 protein level resulted in an advancement of total LDLR protein levels up to 137.1 ± 20.4% and 131.1 ± 25.1%, respectively (Figure 5B).

**Figure 6:**
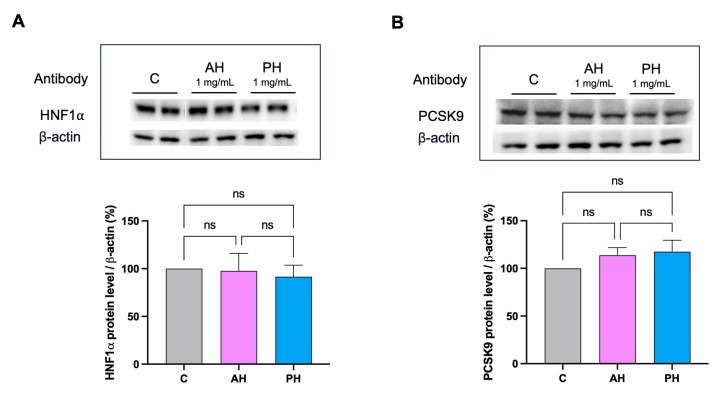
Effects of AH and PH hydrolysates on the PCSK9 pathway. AH and PH extracts did not produce any effect on the modulation of HNF1-α protein levels (**A**) and did not modulate PCSK9 (**B**). Data represent the mean ± s.d. of three independent experiments performed in duplicate. ns: not significant. C: untreated HepG2 cells.

No significant difference was found comparing the two hydrolysates. Both hydrolysates were also unable to modulate the mature PCSK9 protein levels, and they were also ineffective in activating HNF1-α, the PCSK9 transcription factor (Figure 6).

### 3.3. Intestinal Trans-Epithelial Transport of Alcalase (AH) and Papain (AH) Hydrolysates across Caco-2 Cells

**Figure 7:**
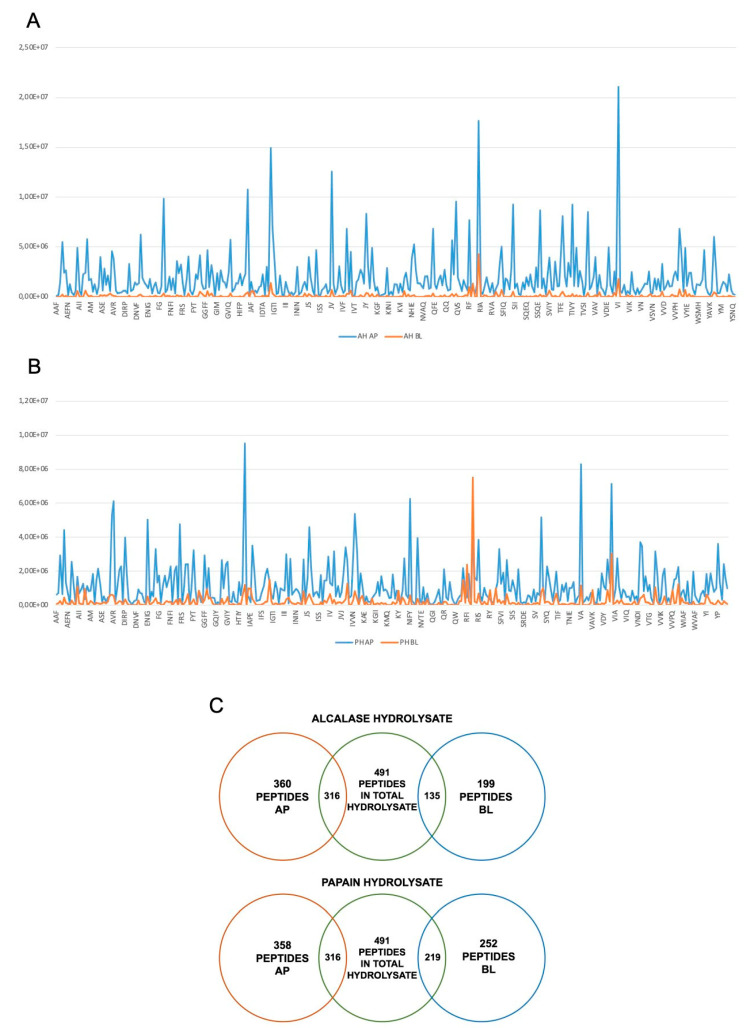
Transport of AH and PH across differentiated Caco-2 cells. Distribution of short-sized peptides in AP and BL compartments for AH (**A**) and PH (**B**), where on the x axis are shown the peptide sequences and on the y axis signal intensity. Schematic description of the peptides absorbed and stable for intestinal absorption (**C**).

Peptides must be absorbed to carry out their biological activity, and, furthermore, they must be resistant to proteolytic hydrolysis given by the action of intestinal proteases and peptidases [36]. In fact, a study on absorption has been carried out to describe the extent to which AH and PH are absorbed by differentiated human intestinal Caco-2 cells. During the experiments, TEER values were monitored. Results indicated that both AH and PH did not alter the intestinal monolayer permeability, as depicted in Figure S1. For AH, 360 peptides in the apical (AP) side and 199 in basolateral (BL) side, whereases for PH, 358 peptides in AP and 252 in BL were identified, respectively. Compared to the original peptide mixture, 316 peptides in AP and 135 peptides in BL were identified for the AH sample. Of these, 134 stable peptides present in both AP and BL were identified for the hydrolysate with alcalase. Regarding the PH sample, 316 peptides in AP and 219 in BL were identified compared to the original mixture. Moreover, the peptides present in both AP and BL were 218 for the papain hydrolysates (Figure 7).

Based on these results, we can state that the sample hydrolyzed with papain contains a greater number of peptides stable for intestinal absorption. The list of peptides absorbed in AP and BL for each hydrolysate and the analysis of stable peptides can be consulted in Supplementary Information. For the AP and BL samples, the percentage of the different peptide species was characterized (Table 1).

In detail, we observed an abundance of di- and tri-peptides in both samples. It is in fact known that small peptides (<3 kDa) are those that show greater biological activity [37]. Furthermore, of the most abundant species in the BL portion in both AH and PH, an analysis was conducted to evaluate their possible toxicity using an in silico method, which is developed to predict and design toxic/non-toxic peptides. No toxicity was predicted for any of the most abundant peptides (Table S1).

## 4. Discussion

### 4.1. Cholesterol Lowering Activity of Olive Seed Hydrolysates

Food-bioactive peptides represent a very dynamic and challenging field of research. It is quite well established that food peptides may exert a plethora of biological effects, including antioxidant, hypocholesterolemic, anti-inflammatory, anti-diabetic, anti-microbial, and hypotensive [33,38]. In this scenario, many efforts have been pursued by the scientific community to foster their health-promoting application; however, it appears clear that some biological effects have been more extensively investigated than some other ones. In this context, cholesterol-lowering peptides are much less studied than antioxidant, anti-diabetic (DPP-IV inhibitory), and/or hypotensive (ACE inhibitory) peptides, respectively [33]. In light of this finding and in an effort to fill this gap, our research activity is mainly focused on the determination and characterization of plant food hydrolysate/peptides with hypocholesterolemic effects. In this study, with the aim of improving a more responsible and sustainable use of food resources, we have focused our interest in valorizing olive seed protein as a new source of food bioactive peptides with the ability to control the metabolism of cholesterol by clarifying their mechanism of action. Olive seed hydrolysates produced with alcalase (AH) and papain (PH) target the activity of both PCSK9 and HMGCoAR, which are two pivotal enzymes involved in the modulation of the cholesterol pathway in hepatic cells [39]. Notably, PCSK9 recognizes and binds LDLR located on the surfaces of hepatic cells, activating receptor catabolism, whereas HMGCoAR, the main target of statins, is involved in the endogenous synthesis of cholesterol [14,39] by hepatic cells.

Findings clearly demonstrated that both AH and PH succeeded in the in vitro impairment of the PCSK9/LDLR PPI with an IC_50_ equal to 0.61 mg/mL and 0.31 mg/mL, respectively (Figure 1C), suggesting that PH is more active than AH. In addition, both hydrolysates inhibited the in vitro HMGCoAR activity with a dose–response behavior, reaching about 50% inhibition at 1 mg/mL. Unlike the effects exerted on the PCK9/LDLR PPI, when comparing the hydrolysates produced with the two different enzymes, no significant difference was observed at the concentration of 1 mg/mL, whereas PH was confirmed to be more active than AH at 0.5 mg/mL (Figure 2C). These results agree with the literature evidence according to which, similarly to AH and PH, lupin protein hydrolysates exert hypocholesterolemic effects targeting in vitro both PCSK9/LDLR PPI and HMGCoAR activity, respectively [15,25]. More in detail, lupin hydrolysates obtained by hydrolyzing the total *L. albus* protein with pepsin and trypsin reduced the PCSK9-LDLR binding by 25% and 23% at 1.0 mg/mL, respectively. In addition, unlike the peptides obtained using pepsin, only tryptic-derived lupin hydrolysate was capable of reducing in vitro HMGCoAR activity with a dose–response trend [25]. Literature evidence suggests that *L. angustifolius* hydrolysate (obtained using alcalase) displayed a similar activity to *L. albus* protein hydrolysate generated using trypsin [40]. In addition, a recent study has reported multiple hempseed protein hydrolysates prepared utilizing pepsin, trypsin, pancreatin, or a combination of these enzymes. The extensive protein cleavage exerted by pancreatin alone or in combination with pepsin leads to the generation of hempseed hydrolysates with limited ability to modulate HMGCoAR activity in vitro, whereas pepsin and trypsin generate hydrolysates with enhanced activity [29]. In addition, our results are in line with other literature evidence. Recently, it was demonstrated that cowpea and amaranth hydrolysates drop HMGCoAR activity [41,42]. Notably, in vitro experiments using the catalytic domain of HMGCoAR demonstrated that the peptides GGV, IVG, and VGVL from amaranth reduced HMGCoAR activity [42]. These findings suggest that these peptides act as HMGCoAR competitive inhibitors, similarly to some peptides from soybean (IAVPGEVA, IAVPTGVA, LPYP, YVVNPDNDEN, and YVVNPDNNEN) and lupin (P5 and P7) [28,43]. However, more detailed studies should be performed to better characterize the hypocholesterolemic mechanism of action at the cellular level and to assess the potential bioavailability of these peptides.

Overall, it appears clear that AH and PH exert HMGCoAR inhibitory activity similarly to hydrolysates from other sources, but they are more active in the impairment of the PCSK9 ability to bind the LDLR than lupin hydrolysates. This enhanced dual inhibitory activity makes AH and PH two innovative cholesterol-lowering peptide mixtures that, through the inhibition of both PCSK9 and HMCoAR activity, modulate intracellular cholesterol metabolism at the cellular level. More specifically, it was shown that both AH and PH increased the levels of the LDLR protein on the surface of human hepatic cells, which improved HepG2 cells’ capacity to absorb LDL from the extracellular environment from a functional point of view (Figure 4A,B). Dedicate experiments confirmed that both AH and PH restored the PCSK9-reduced membrane LDLR protein level and functionality, renewing the ability of HepG2 to improve the PCSK9-reduced LDLR absorption (Figure 4C,D). To better elucidate the molecular mechanism of action through which these peptides exert an in vitro hypocholesterolemic effect, it was demonstrated by western blotting analysis that both AH and PH increased the total LDLR protein levels through the activation of the SREBP-2 transcription factor (Figure 5A,B). Interestingly, AH and PH were not able to modulate HNF1-α, therefore being ineffective in modulating PCSK9 protein levels. These results are in line with the behavior of hempseed hydrolysates obtained using pepsin, and they are different from that of lupin hydrolysates obtained using pepsin as well. Indeed, unlike lupin peptides (AH and PH), the hempseed peptide mixture improved the PCSK9 protein level through the modulation of HNF1-α, displaying a statin-like mechanism of action [27].

### 4.2. Assessment of Trans-Epithelial Transport of AH and PH Peptide Mixtures Using Differentiated Caco-2 Cells

In the field of food bioactive peptides, a significant challenge to their rapid utilization as nutraceuticals and functional foods is their limited metabolic stability and bioavailability at the intestinal level [44]. The first physiological barrier that dietary peptides and hydrolysates meet is the intestinal brush boundary. There are two primary events that may occur when these molecules come into contact with human enterocytes: certain peptides get transported by the cells, while others become metabolized inside the cellular milieu. It is important to underline that the metabolic degradation of bioactive peptides does not necessarily imply a loss of bioactivity [7].

Due to its direct impacts on the chemical compositions and peptidomic profiles of protein hydrolysates and peptides, the highly complex intestinal environment actively regulates their bioactivity. Consequently, conducting in vitro experiments to simulate the intestinal transport of food protein hydrolysates is crucial before proceeding with expensive in vivo experiments to confirm their health-promoting activities. In this context, human intestinal Caco-2 cells are commonly used as an effective model for in vitro evaluation of the propensity of food bioactive peptides to be transported by intestinal cells. In this study, differentiated Caco-2 cells were incubated with both AH and PH (at a concentration of 1 mg/mL) on the apical side for 2 h. The apical (AP) and basolateral (BL) solutions were then collected and analyzed by UHPLC-HRMS. The analysis of short-sized peptides clearly indicated a different peptidomic profile in the AP and BL samples (Figure 7A,B). In more detail, first, different peptides were determined in the AH and PH, confirming that enzyme activity is crucial for the determination of peptide mixture composition. Thus, when these two mixtures encountered the brush border level, intestinal cells selectively modulated the transport of specific peptides rather than other ones. This is clearly highlighted in Figure 7A,B, where it is doubtless depicted that all the short-sized peptides display lower signal intensity in the BL solution than in the AP one; however, peptides from PH are higher transported by Caco-2 cells than AH peptides. Notably, RFI, VI, RIA, and IG from the AH mixture and RI, RFI, RE, RI, SFVI, and VIA from the PH mixture are the peptides mainly transported by differentiated Caco-2, interestingly suggesting that the R residue in the N-terminus of the short peptides plays an important role in their in vitro bioavailability.

In the gastrointestinal tract, mature enterocytes produce microvilli, which serve as the main surface for nutrition absorption. These microvilli membranes contain enzymes that facilitate the breakdown of complex nutrients into simpler compounds that can be absorbed more easily. From a physiological perspective, the dynamic balance between bioactive peptide transport and degradation is essential. Differentiated Caco-2 models are reliable tools for studying the proteolytic activity of the brush border barrier, as they express a wide range of membrane peptidases, including DPP-IV and ACE, on the apical side of enterocytes. In light of this theory, our results show that out of the total 491 short peptides identified in the AH and PH hydrolysates, 316 are present in the AP solutions, suggesting that about 64.4% of peptides, which were present in the original AH sample, are stable at the metabolic degradation exerted by the apical side of intestinal cells, and less than 40% are metabolized by active intestinal peptidases (Figure 7C). In addition, out of the 199 and 258 AH and PH peptides identified in the BL solution, 135 and 219 peptides were in common with total AH and PH, respectively (Figure 7C). These results clearly suggested that 27.5% and 44.6% of the short-sized peptides identified in the AH and PH samples, respectively, were intactly transported by intestinal cells (Figure 7C). For both the AH and PH samples, tripeptides are the most abundant moiety. In detail, in the AP portion, they correspond to 42.5% in AP and 38% in BL for the AH sample, and they correspond to 42.1% in AP and 41.3% in BL for the PH sample (Table 1). In general, food-derived peptides can be transported across the intestinal brush-border membrane into the bloodstream through several routes: (i) peptide transport 1 (PepT1)-mediated route, (ii) paracellular route via tight junctions (TJs), (iii) transcytosis route, and (iv) passive transcellular diffusion. The absorption of peptides through these routes is influenced by factors such as peptide size, charge, hydrophobicity, and degradation by peptidases. Short peptides, such as dipeptides and tripeptides, are preferentially transported by PepT1, which is highly expressed in the intestinal epithelium. Highly hydrophobic peptides, on the other hand, are transferred by transcytosis or passive transcellular diffusion. During the experiments, the TEER of the intestinal monolayer was measured, and no effect on cell permeability was detected, suggesting that both AH and PH do not affect the monolayer permeability. In addition, using the ToxinPred database, it was confirmed that the most abundant species in the BL portion of both AH and PH do not display a toxic effect using this in silico system (Table S1). Overall, this in silico output is in agreement with the MTT experiments on HepG2 and Caco-2 cells [5] and with functional TERR measurement.

## 5. Conclusions

In conclusion, using our multidisciplinary approach, we have provided new insights that reinforce the evidence supporting the multifunctional behavior of food bioactive hydrolysates from a new plant source, clearly suggesting that they might be beneficially utilized as novel ingredients in the creation of dietary supplements intended to prevent metabolic syndrome. In addition, based on our findings, the general critical issues related to the low bioavailability and intestinal stability of food peptides were successfully faced, providing the peptidomic profile of short-sized peptides able to be stablely transported by in vitro human intestinal cells. This study may be considered an important starting point for further investigation of their health-promoting effects using an animal model.

## Figures and Tables

**Table 1:** Percentage of the different peptide species.

Peptide Length (AA).	Abudance (%)	Sample
2	16.1	AH AP
	31.7	AH BL
	17.0	PH AP
	22.2	PH BL
3	42.5	AH AP
	37.9	AH BL
	42.1	PH AP
	41.3	PH BL
4	38.0	AH AP
	28.0	AH BL
	37.6	PH AP
	33.3	PH BL
5	3.4	AH AP
	2.5	AH BL
	3.3	PH AP
	3.2	PH BL

## Data Availability

Data are contained within the article.

## Supplementary Materials

The following supporting information can be downloaded at: https://www.mdpi.com/article/10.3390/nu16030371/s1, Detailed description of material and methods, Table S1: Representation of the toxicity prediction obtained using ToxinPred and of the potential bioactivity by BIOPEP, Figure S1: Time course of TEER changes recorded in untreated (control), AH, and PH-treated Caco-2 cells. Table AP-BL, in which the identified peptide sequences are listed.
