# Supplementary material for: Olive (Olea europaea L.) Seed as New Source of Cholesterol-Lowering Bioactive Peptides: Elucidation of Their Mechanism of Action in HepG2 Cells and Their Trans-Epithelial Transport in Differentiated Caco-2 Cells
Source: Nutrients. 2024 Jan 26;16(3):371. doi: 10.3390/nu16030371 (PMC10857614; doi:10.3390/nu16030371)
Supplement: Supplementary file 1 [file nutrients-16-00371-s001.zip › nutrients-2834392-supplementary.pdf]

Supplementary Materials

Olive (*Olea europaea* L.) Seed as New Source of Cholesterol-Lowering Bioactive Peptides: Elucidation of Their Mechanism of Action in HepG2 Cells and Their Trans-Epithelial Transport in Differentiated Caco-2 Cells

Martina Bartolomei [1], Jianqiang Li [1], Anna Laura Capriotti [2], Melissa Fanzaga [1], Lorenza d'Adduzio [1], Aldo Laganà [2], Andrea Cerrato [2], Nadia Mulinacci [3], Lorenzo Cecchi [4], Carlotta Bollati [1] and Carmen Lammi [1,*]

[1] Department of Pharmaceutical Sciences, University of Milan, 20133 Milan, Italy; martina.bartolomei@unimi.it (M.B.); melissa.fanzaga@unimi.it (M.F.); lorenza.dadduzio@unimi.it (L.d.)

[2] Department of Chemistry, Sapienza University of Rome, Piazzale Aldo Moro 5, 00185 Rome, Italy; aldo.lagana@uniroma1.it (A.L.); andrea.cerrato@uniroma1.it (A.C.)

[3] Department of Neuroscience, Psychology, Drug and Child Health, Pharmaceutical and Nutraceutical Section, University of Florence, 50019 Florence, Italy; nadia.mulinacci@unifi.it

[4] Department of Agricultural, Food, Environmental and Forestry Sciences and Technologies, University of Florence, Via Donizetti, 50144 Florence, Italy; lo.cecchi@unifi.it

* Correspondence: carmen.lammi@unimi.it; Tel.: +39-02-50319372

2. Materials and Methods

2.1. Chemicals

Dulbecco's modified Eagle's medium (DMEM), stable L-glutamine, fetal bovine serum (FBS), phosphate buffered saline (PBS), penicillin/streptomycin, chemiluminescent reagent, 24 and 96-well plates were purchased from Euroclone (Milan, Italy). The Transwell, polycarbonate filters (12 mm diameter, 0.4 μ m pore diameter) were purchased from Corning Inc. (Lowell, MA, US). 3 kDa cut-off Millipore UF System ultrafiltration was purchased from membrane (Millipore, Bedford, MA, USA). The HMGCoAR assay kit, bovine serum albumin (BSA), MTT [3-(4,5-dimethylthiazol-2-yl)-2,5-diphenyltetrazolium bromide], Janus Green B, formaldehyde, HCl and H_2SO_4 were from Sigma-Aldrich (St. Louis, MO, USA). Antibody against LDLR and the 3,3', 5,5' -tetramethylbenzidine (TMB) substrate were bought from Thermo Fisher Scientific (Waltham, MA, USA). The LDL-DyLightTM 550 was from Cayman Chemical (Ann Arbor, MI, USA). The CircuLex PCSK9 in vitro binding Assay Kit was from CircuLex (CycLex Co., Nagano, Japan). Phenylmethanesulfonyl fluoride (PMSF), Na-orthovanadate inhibitors, and the antibodies against rabbit Ig-horseradish peroxidase (HRP), mouse Ig-HRP, and SREBP-2 were purchased from Santa Cruz Biotechnology Inc. (Santa Cruz, CA, USA). The antibodies against hepatocyte nuclear factor 1-alpha (HNF1-alpha) and PCSK9 were bought from GeneTex (Irvine, CA, USA). The inhibitor cocktail Complete Midi was from Roche (Basel, Switzerland). Mini protean TGX pre-cast gel 7.5% and Mini nitrocellulose Transfer Packs were purchased from BioRad (Hercules, CA, USA).

2.2. Sample Preparation

Alcalase and papain olive seed hydrolysates were prepared as previously described [5]. Briefly, olive seeds were grounded with a domestic mill and hexane (ratio 1:20 w/v) was used to defatten olive seed powder for 1h while magnetic stirring was in place. After drying, the defatted powder was subjected to protein extraction. In detail, 1.5 g of defatted powder were mixed with 30 mL of extracting solution containing UREA 6 M, 0.1 M Tris-HCl (pH 8), 0.5 M NaCl, 0.5% SDS, and 0.1% DTT. After extraction from the olive seed, proteins were hydrolyzed with Alcalase (50°C, 4h, 0.15 UA/g, pH 8.5) and Papain (65°C, 8h, 100 UA/g, pH 7) enzymes. Both hydrolysates were ultrafiltrated with 3 kDa cut-off Millipore UF System ultrafiltration membrane using optimized conditions [7].

2.3. Cell Culture

Human hepatic HepG2 cells (ATCC, HB-8065, ATCC from LGC Standards, Milan, Italy) and human intestinal Caco-2 cells INSERM (Paris, France) were cultured in DMEM high glucose with stable L-glutamine, supplemented with 10% FBS, 100 U/mL penicillin, 100 μg/mL streptomycin (complete growth medium) with incubation at 37 °C under 5% CO_2 atmosphere.

2.4. 3-(4,5-Dimethylthiazol-2-yl)-2,5-Diphenyltetrazolium Bromide (MTT) Assay

A total of 3 × 10^4 HepG2 cells/well were seeded in 96-well plates and treated with 0.1, 0.5, 1.0, 1.5 and 5.0 mg/mL of AH and PH samples, or vehicle (H_2O) in complete growth media for 48 h at 37 °C under 5% CO_2 atmosphere. MTT experiments have been performed following conditions already optimized [21]. Subsequently, the treatment solvent was aspirated and 100 μL/well of 3-(4,5- dimethylthiazol-2-yl)-2,5-diphenyltetrazolium bromide (MTT) filtered solution added. After 2 h of incubation at 37 °C under 5% CO_2 atmosphere, 0.5 mg/mL solution was aspirated and 100 μL/well of the lysis buffer (8 mM HCl + 0.5% NP-40 in DMSO) added. After 5 min of slow shaking, the absorbance at 575 nm was read on the Synergy H1 fluorescence plate reader (Biotek, Bad Friedrichshall, Germany).

2.5. In Vitro PCSK9-LDLR Binding Assay

Alcalase and Papain hydrolysates (1 mg/mL) were tested using the *in vitro* PCSK9-LDLR binding assay (CycLex Co., Nagano, Japan) following the manufacture instructions and conditions already optimized [22]. Briefly, plates are pre-coated with a recombinant LDLR-AB domain, which contains the binding site for PCSK9. The vehicle and/or tested peptides were added to microcentrifuge tubes and diluted in reaction buffer prior to the experiment commencing. Afterwards, the reaction mixtures were added in each well of the microplate and the reaction was started by adding His-tagged PCSK9 wild type solution (3 μl). The microplate was allowed to incubate for 2h at RT shaking at 300 rpm on an orbital microplate shaker. The biotinylated anti-His-tag monoclonal antibody (100 μl) was added and incubated at RT for 1h shaking at 300 rpm. After incubation, wells were washed for 4 times with wash buffer. After the last wash, 100 μl of HRP-conjugated streptavidin were added and the plate was incubated for 20 min at RT. After incubation, wells were washed 4 times with wash buffer. Finally, the substrate reagent was added and the plate was incubated for 10 min at RT shaking at ca. 300 rpm. The reaction was stopped with 2.0 N sulfuric acid and the absorbance at 450 nm was measured using the Synergy H1 (Biotek, Bad Friedrichshall, Germany).

2.6. In-Cell Western (ICW) Assay

The ICW assay was performed using the same previously optimized procedure [23]. Briefly, a total of 3 × 10^4 HepG2 cells/well were seeded in 96-well plate and, the following day, they were treated with 4.0 μg/mL PCSK9-WT, 1 mg/mL of Alcalase and Papain hydrolysates, 4.0 μg/mL PCSK9 + 1 mg/mL of Alcalase and Papain hydrolysates and vehicle (H_2O) for 2 h at 37 °C under 5% CO_2 atmosphere. Subsequently, they were fixed in 4% paraformaldehyde for 20 min at RT. Cells were washed 5 times with 100 μL of PBS/well and the endogenous peroxides activity quenched adding 3% H_2O_2 for 20 min at RT. Non-specific sites were blocked with 100 μL/well of 5% BSA in PBS for 1.5h at RT. LDLR primary antibody solution (Abcam) (1:3000 in 5% BSA in PBS, 25 μL/well) was incubated O/N at +4 °C. Afterwards, the primary antibody solution was discarded, and each sample was washed 5 times with 100 μL/well of PBS. Goat anti-rabbit Ig-HRP secondary antibody (Santa Cruz) solution (1:6000 in 5% BSA in PBS, 50μL/well) was added and incubated 1h at RT. The secondary antibody solution was washed 5 times with 100 μL/well of PBS (each wash for 5 min at RT). Fresh prepared TMB Substrate (Pierce, 100 μL/well) was added, and the plate was incubated at RT until the desired color was developed. The reaction was stopped with 2 M H_2SO_4 and then the absorbance at 450 nm was measured using a microplate reader Synergy H1 from Biotek. Cells were stained by adding 1 × Janus green stain, incubating for 5 min at RT. The dye was removed, and the sample washed 5 times with water. Afterward, 0.1 mL 0.5 M HCl per well were added and incubated for 10 min. After 10 s shaking, the OD at 595 nm was measured using the Synergy H1 fluorescent plate reader from Biotek.

2.7. Fluorescent LDL Uptake

LDL Uptake assay was carried out following condition already described [24]. Shortly, a total of 3 × 10^4 HepG2 cells/well were seeded in 96-well plates and then kept in complete growth medium for 2 d before treatment. On the third day, cells were treated with 4.0 μg/mL PCSK9-WT, 1 mg/mL of Alcalase and Papain hydrolysates, 4.0 μg/mL PCSK9 + 1 mg/mL of Alcalase and Papain hydrolysates and vehicle (H_2O) for 2 h at 37 °C under 5% CO_2 atmosphere. At the end of the treatment periods, the culture medium was replaced with 75 μL/well LDL-DyLight 549 working solution. The cells were additionally incubated for 2 h at 37 °C, and then the culture medium was aspirated and replaced with PBS 100 μL/well. The degree of LDL uptake was

measured using the Synergy H1 fluorescent plate reader from Biotek (excitation and emission wavelengths 540 and 570 nm, respectively).

2.8. Western Blot Analysis

Immunoblotting experiments were performed using optimized protocol [25]. A total of 1.5 × 10^5 HepG2 cells/well (24-well plate) were treated with 1 mg/mL of AH and PH for 24 h. After each treatment, the supernatants were collected and stored at -80 °C. After cell lysis the protein concentration was evaluated by the Bradford's method and 50 μg of total proteins loaded on a precast 7.5% Sodium Dodecyl Sulfate-Polyacrylamide (SDS-PAGE) gel at 130 V for 45 min. Subsequently, the gel was pre-equilibrated in H_2O for 5 min at room temperature (RT) and transferred to a nitrocellulose membrane (Mini nitrocellulose Transfer Packs,) using a Trans-Blot Turbo at 1.3 A, 25 V for 7 min. Target proteins, on milk or BSA blocked membrane, were detected by primary antibodies as follows: anti-SREBP-2, anti-LDLR, anti-HNF1a, anti-PCSK9 and anti-β-actin. Secondary antibodies conjugated with HRP and a chemiluminescent reagent were used to visualize target proteins and their signal was quantified using the Image Lab Software (Biorad, Hercules, CA, USA). The internal control β-actin was used to normalize loading variations.

2.9. HMGCoAR A activity Assay

The experiments were conducted following the manufacturer instructions and optimized protocol [26]. The assay buffer, NADPH, substrate solution, and HMGCoAR were provided in the HMGCoAR Assay Kit. Specifically, each reaction (200 μL) was set up adding the reagents accordance with the following procedure: 1× assay buffer, a 0.1 and 0.5 mg/mL of Alcalase and Papain hydrolysate or vehicle (C), the NADPH (4 μL), the substrate solution (12 μL), and lastly the HMGCoAR (catalytic domain) (2 μL). Eventually, the samples were mixed and the absorbance at 340 nm read by the microplate reader Synergy H1 at time 0 and 10 min. The HMGCoAR-dependent oxidation of NADPH and the inhibition properties of peptides were measured by absorbance reduction, which is directly proportional to enzyme activity.

2.10. Caco-2 Cell Culture and Differentiation

Caco-2 cells were cultured as reported by a previous protocol [27]. For differentiation, cells were seeded on a Transwell at 3.5 × 10^5 cells/cm^2 density in complete medium supplemented with 10% FBS in both AP and BL compartments for 2 d to allow the formation of a confluent cell monolayer. Starting from day three after seeding, cells were transferred to FBS-free medium in the AP compartment and allowed to differentiate for 18-21 days with regular medium changes three times weekly [28]. To monitor the cell monolayers integrity, the transepithelial electrical resistance (TEER) of differentiated Caco-2 cells was estimated at 37 °C using the voltmeter apparatus Millicell (Millipore Co., Billerica, MA, USA), promptly before and at the end of the transport tests.

2.11. Trans-Epithelial Transport Experiments

Prior to experiments, the cell monolayer integrity and differentiation were checked by TEER measurement. Hydrolysates trans-epithelial passage was assayed in differentiated Caco-2 cells in transport buffer solution (137 mM NaCl, 5.36 mM KCl, 1.26 mM CaCl2, and 1.1 mM MgCl2, 5.5 mM glucose) according to previously described conditions [29]. With the purpose of reproduce the pH conditions existing in vivo in the small intestinal mucosa, the apical (AP) solutions were maintained at pH 6.0 (buffered with 10 mM morpholinoethane sulfonic acid), and the basolateral (BL) solutions were maintained at pH 7.4 (buffered with 10 mM N-2-hydroxyethylpiperazine-N-4-butanesulfonic acid). Prior to transport experiments, cells were washed twice with 500 μL PBS containing Ca++ and Mg++. Alcalase and papain hydrolysates (1 mg/mL) where added in the AP compartment in the AP transport solution (500 μL) and the BL compartment with the BL transport solution (700 μL). After 2h at 37°C, all BL and AP solutions were collected and were stored at -80 °C prior to analysis. Three independent transport experiments were performed, each in duplicate.

2.12. UHPLC-HRMS Analysis and Short-Sized Peptide Identification

Short peptides were analyzed by Vanquish binary pump H (Thermo Fisher Scientific) coupled to a hybrid quadrupole- Orbitrap mass spectrometer Q Exactive (Thermo Fisher Scientific) using a heated ESI source operating in positive ion mode. The mass-spectrometric strategy was set up as previously reported

[30]. Each sample (20 µL) was injected onto a Kinetex XB-C18 (100 × 2.1 mm, 2.6 µm particle size, Phenomenex, Torrance, USA). Chosen flow, column temperature, and gradient parameters are reported in our previous work without any modification [31]. Untargeted suspect screening analysis was performed in the top 5 data-dependent acquisition mode in the range m/z 150-750 with a resolution (full width at half maximum, FWHM, m/z 200) of 70,000. Higher-energy collisional dissociation fragmentation was performed at 40% normalized collision energy at the resolution of 35,000 (FWHM, m/z 200). An inclusion list, in which all precursor ions deriving from the combination of the 20 amino acids in di-, tri-, and tetrapeptides were listed, was included in the method [30]. Raw data files from three experimental replicates and a blank sample were processed by Compound Discoverer using a workflow specifically dedicated to short peptide analysis. The database of short peptide sequences with IDs, masses, and molecular formulas, was implemented for the automatic matching of the extracted m/z ratios. Extracted masses from the chromatograms were aligned and filtered to remove background compounds present in the blank sample, features whose masses were not present in the databases, and those not fragmented. Filtered features were manually validated, matching experimental spectra to those generated in silico by mMass 5.5 [32].

2.13. In Silico Toxicity Prediction of the Bioavailable Fraction of AH and PH Hydrolysates

The peptides which were identified as the most abundant in the BL side of the Transwell system exploited for the absorption studies for both AH and PH hydrolysates, were analyzed using a web-based server Toxinpred (https://webs.iiitd.edu.in/raghava/toxinpred/multi_submit.php), useful to identify and predict highly toxic or non-toxic peptides from large number of submitted sequences in FASTA format [33].

2.14. Statistical Analysis

All measurements were performed in triplicate, and results were expressed as the mean ± standard deviation (s.d.), where p-values < 0.05 were considered to be significant. Statistical analyses were performed by one-way ANOVA followed by Dunnett’s and Tukey’s post-test (Graphpad Prism 9, GraphPad Software, La Jolla, CA, USA).

Table S1 Representation of the toxicity prediction obtain using ToxinPred and of the potential activity using BioPep.ACE: angiotensin-convertin enzyme; DPP: dypeptidil-dipeptidase; HMGCoAR: 3-hydroxy-3-methylglutaryl coenzyme A Reductase; antiox: antioxidant activity.

SAMPLE	PEPTIDE SEQUENCE	PREDICTION	BIOPEP
**AH**	AI	Non-Toxin	ACE inhibitor
	APA	Non-Toxin	ACE and DPPIV inhibitor
	IGEE	Non-Toxin	ACE and DPPIV inhibitor
	IVR	Non-Toxin	ACE and DPPIV inhibitor
	RF	Non-Toxin	ACE and DPPIV inhibitor
	RFI	Non-Toxin	ACE and DPPIV inhibitor
	IV	Non-Toxin	Glucose Uptake stimulating
	RI	Non-Toxin	DPP-IV inhibitor
	SFI	Non-Toxin	ACE, renin, and DPPIV inhibitor
	SVIY	Non-Toxin	ACE and DPPIV inhibitor and antiox
	VI	Non-Toxin	DPPIV inhibitor
	VVPQ	Non-Toxin	ACE and DPPIV inhibitor
	VY	Non-Toxin	ACE and DPPIV inhibitor and antiox

**PH**	RF	Non-Toxin	ACE and DPPIV inhibitor
	RI	Non-Toxin	DPP-IV inhibitor
	IVR	Non-Toxin	ACE and DPPIV inhibitor
	RFI	Non-Toxin	ACE and DPPIV inhibitor
	IGEE	Non-Toxin	ACE and DPP-IV and III inhibitor
	IAPE	Non-Toxin	ACE and DPP-IV and III, and alpha glucosidase inhibitor
	SVIY	Non-Toxin	ACE and DPPIV inhibitor and antiox
	VY	Non-Toxin	ACE and DPPIV inhibitor and antiox
	AIPA	Non-Toxin	ACE and DPPIV inhibitor and antiox
	SFI	Non-Toxin	ACE, renin, and DPPIV inhibitor
	VA	Non-Toxin	DPP-IV inhibitor
	AI	Non-Toxin	ACE inhibitor
	IA	Non-Toxin	ACE inhibitor
	IAF	Non-Toxin	HMGCoAR, ACE and DPP-IV inhibitor
	VV	Non-Toxin	DPPIV inhibitor
	VI	Non-Toxin	DPPIV inhibitor

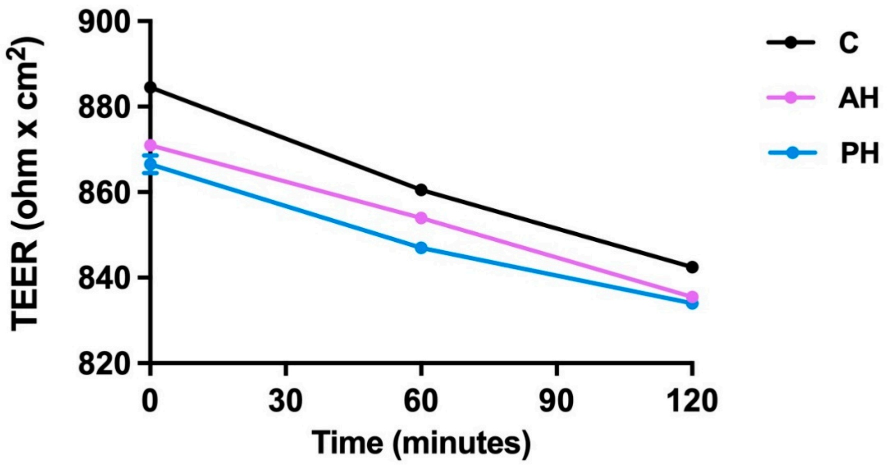

Figure S1: Time course of TEER changes recorded in untreated (control), AH, and PH-treated Caco-2 cells.
